# Influence of Pasteurisation (Conventional vs. Radiofrequency) and Chill Storage on Retention of Ascorbic Acid, Tocopherol and Carotenoids in Salmorejo

**DOI:** 10.3390/foods13020349

**Published:** 2024-01-22

**Authors:** Marina Kravets, Francisco Javier García-Alonso, Andrés Abea, Maria Dolors Guàrdia, Israel Muñoz, Sancho Bañón

**Affiliations:** 1 Department of Food Technology and Science and Nutrition, Veterinary Faculty, Regional Campus of International Excellence “Campus Mare Nostrum”, University of Murcia, 30100 Murcia, Spain; marina.kravets@um.es (M.K.); fjgarcia@um.es (F.J.G.-A.); 2 Institut de Recerca i Tecnologia Agroalimentàries IRTA—Food Technology Program, Finca Camps i Armet, Monells, 17121 Girona, Spain; andres.abea@irta.cat (A.A.); dolors.guardia@irta.cat (M.D.G.); israel.munoz@irta.cat (I.M.)

**Keywords:** ascorbic acid, tocopherol, carotenoids, thermal, oxidative, stability, tomato-oil puree

## Abstract

Salmorejo, a Mediterranean tomato-oil puree, is considered a dietary source of antioxidant vitamins C and E and carotenoids lycopene and β-carotene, the latter endowed with provitamin A activity. However, these antioxidants can be degraded in oxidation reactions catalysed or not by enzymes during pasteurisation and storage treatments used to stabilise the salmorejo before consumption. Due to its better penetration, the use of dielectric heating by radiofrequency (RF) may improve results of pasteurisation in this product. The objective was to assess the effects of pasteurisation temperature (70–100 °C, at 5 °C intervals) and storage time (0–5 months, at one-month intervals) on levels of ascorbic acid, α-tocopherol and carotenoids and antioxidant capacity (AC) in salmorejo pasteurised (over 10 s) by conventional (CH) or RF continuous heating. Two successive experiments were conducted to select an adequate pasteurisation temperature for use in the shelf-life study. Pasteurisation upon tested conditions allows a good retention of salmorejo antioxidants. Either CH or RF pasteurisers can be used with similar results. Vitamin C (L-ascorbic + dehydroascorbic acids) was more abundant (15–19 mg 100 g^−1^) than carotenoids (0.9–2.6 mg 100 g^−1^) (all-*trans* + *cis* lycopene and β-carotene) and α-tocopherol (0.8–1.2 mg 100 g^−1^) in the pasteurised product. Using excessively low temperatures (70 °C) resulted in partial losses of the three antioxidants, possibly due to oxidase residual activities. Intensifying thermal treatment improved this issue with minor losses of the thermolabile vitamin C and increased carotenoid content. Using a suitable temperature (80 °C) did not prevent most vitamin C from being degraded by the first month, while α-tocopherol, and, to a lesser extent, carotenoids, showed good retention levels during shelf life under refrigeration. Vitamins C and E and carotenoids, either by degradation, regeneration or releasing, likely contribute to the AC in salmorejo. Phenolic antioxidant response, radical-scavenging activities and redox potential values confirmed this finding. The pasteurised-chilled salmorejo shows good antioxidant properties with potential health implications, a positive nutritional aspect for consumers of this tomato-oil homogenate. The losses of antioxidants and AC due to pasteurization would be of little relevance compared to the losses accumulated during shelf life.

## 1. Introduction

The consumption of tomato products has been reported as having excellent antioxidant, anti-inflammatory and anticancer properties [1], hence the interest in applying elaboration methods that enable retention of the maximum content of bioactive compounds. Consumers expect tomato products to provide relevant levels of healthy antioxidants; however, antioxidants can often be degraded during processing and shelf life. This might occur with salmorejo, a Spanish cold purée prepared with whole tomatoes, extra virgin olive oil (EVOO), bread, vinegar, garlic and salt, whose consumption and international trade is on the rise [2]. Industrial salmorejo is aerobically homogenised to obtain a creaming puree, pasteurised with “High Temperature Short Time” (HTST) treatments, and packed in bricks or plastic bottles. Salmorejo can reach a shelf life under refrigeration of up to six months as it is a microbiologically stable acidic product [3], though tends to develop undesirable changes by oxidation, so that pasteurisation must be carefully conducted to inactivate oxidase enzymes without altering thermolabile components, including antioxidants. Therefore, the use of dielectric heating by radiofrequency (RF) has recently been proposed to improve the results of pasteurisation in this type of tomato-oil emulsions [4]. Compared to microwave (MW) heating, the most extended technology, RF heating works at lower frequencies (10–300 MHz) and enables wave energy to penetrate more deeply into the product, obtaining more uniform heating [5]; however, the power output is lower in RF than in MW heating systems. Thus, larger RF systems are required to obtain the same power rating, and slower heating rates are achieved with RF than with MW [6]. RF heating (27.12 MHz) is demonstrated to have a positive impact on the nutritional quality of cooked tomato puree by preserving endogenous antioxidants (L-ascorbic acid) and colour characteristics [7].

Salmorejo can be considered a dietary source of bioavailable antioxidants from tomato and EVOO (L-ascorbic acid, α-tocopherol, lycopene and carotene) [8,9]. L-ascorbic acid is a radical oxygen species (ROS) scavenger responsible for the primary antioxidant mechanism in fruits and vegetables. A rapid degradation of L-ascorbic acid can be used as a reliable oxidation index in fruit products sensitive to heat, light and oxygen [10]. L-ascorbic acid can be reversibly oxidised to dehydroascorbic acid upon exposure to light, heat, transition metal ions and pH (alkaline condition), then dehydroascorbic acid further irreversibly hydrolyses to form 2,3-diketogulonic acid [11]. Both L-ascorbic and dehydroascorbic acids are deemed different chemical species of vitamin C. α-tocopherol is a natural antioxidant from vegetable oils that can also be present in tomatoes [12]. EVOO contains more α-tocopherol (over 200 mg kg^−1^) than β- and γ-tocopherol (<15 mg kg^−1^), contributing with its antioxidant activities to thermal stability [13]. α-tocopherol inhibits formation of hydroperoxides and oxidised secondary lipids (e.g., aldehydes, ketones, and alcohols) from unsaturated lipids [14]. Lycopene and β-carotene, the main natural pigments of tomatoes, are carotenoids with a similar chemical structure and molecular weight. β-carotene is formed from all-*trans* lycopene in a reaction catalysed by the lycopene cyclase enzyme. Lycopene is a linear molecule, has more conjugated double bonds and presents more antioxidant activity than β-carotene [15]. Cooking procedures are required to produce relevant losses of carotenoids in tomato products [16,17,18,19]. Carotenoids can also degrade by oxidation reactions catalysed or not by enzymes, which depend on the availability of oxygen and carotenoid structure; oxidation is stimulated by light, heat, some trace metals, enzymes and peroxides and is inhibited by antioxidants [20]. Several studies reported that vitamin C and carotenoids reasonably resist HTST treatments used to stabilise tomato products, such as vegetable beverages (90–98 °C, 15–21 s) [21] and purees (70–108 °C, 35–60 s) [22,23,24]. Tocopherols also remain relatively undegraded in the pasteurised tomato products and may even concentrate with moisture loss [25]. Many of the above-mentioned studies agree that antioxidant capacity (AC) is positively related with levels of antioxidants retained intact by tomato products. The hydrophilic fraction contributes more AC than the lipophilic fraction when both are separately studied in tomato products [26,27,28].

A recent study revealed that the microbiological quality of salmorejo can be easily ensured with HTST pasteurisation treatments, though the temperature applied, either by defect or by excess, can favour enzyme oxidation or produce overheating signs, respectively [29]. A further study [30] confirmed that the shelf life of pasteurised-chilled salmorejo is mainly limited by long-term oxidation involving some deterioration signs (discolouration, flavour alteration, shear thinning and lipid oxidation). In both studies, enzyme residual activities were crucial for oxidative stability in salmorejo; the relative activities of the polyphenol oxidase (PPO) and peroxidase (POD) enzymes decreased from 70% to less than 10% depending on the pasteurisation temperature used, while drastically diminished (<5%) after one month in refrigeration. Considering the above, the research hypothesis was that the retention of key antioxidants (vitamin C and E, and carotenoids) in salmorejo may vary under different pasteurisation and storage conditions. The first objective was to know what temperatures (70–100 °C) improve retention levels of ascorbic acid, α-tocopherol and carotenoids and resulting AC in salmorejo pasteurised by conventional heating (CH) or RF. The second objective was to determine the stability of these antioxidants and AC during the shelf life (up to five months) of salmorejo pasteurised at one optimised temperature.

## 2. Materials and Methods

### 2.1. Experimental Design

Antioxidant properties were considered to assess pasteurisation results for a salmorejo alternatively treated with conventional or RF heating. Two successive experiments were conducted for this purpose. In the pasteurisation experiment, antioxidants (vitamin C, α-tocopherol and carotenoids) and AC (assessed by four different assays) were studied in salmorejo pasteurised with CH or RF at increasing temperatures (70–100 °C). From the results of the above experiment, a pasteurisation temperature was selected to study antioxidant properties in the product kept at refrigeration. In the shelf-life experiment, antioxidants and AC were again studied during the shelf life (0–5 months) of salmorejo pasteurised with CH or RF at 80 °C. Raw samples were also analysed in both experiments.

### 2.2. Salmorejo Manufacturing Process

Salmorejo ingredients (g 100 g^−1^) were: whole vine tomatoes (87.6), EVOO (Var. Hojiblanca) (5.0), breadcrumbs (3.0), inulin (Orafti^®^ HP, Beneo, Barcelona, Spain) (2.0), wine vinegar (1.5), garlic (0.7) and salt (0.2). Briefly, tomatoes were crushed and sieved (3 mm diameter). The resulting purée was then mixed with the rest of ingredients in a stirring tank for 30 min. Raw salmorejo was homogenised in a MZ-100 colloid mill (FrymaKoruma AG, Rheinfelden, Switzerland) and preheated at 50 °C for 15 s in a tubular heat exchanger. Before pasteurisation, preheated product was homogenised at 250 bars (HA31002, Bertoli SRL, Parma, Italy). Pasteurisation was conducted to inactivate *E. coli* O157:H7, *S. enterica* and *L. monocytogenes* to ensure at least a 5-log reduction of vegetative pathogens of concern. Salmorejo was heated at 70–100 °C (at 5 °C intervals) in a continuous pasteurisation line (200 L product h^−1^) using a CH (2-stage tubular heat exchanger, Inoxpa, Banyoles, Barcelona, Spain) or a RF equipment (45 kW EVO RF Cartigiliano, Vicenza, Italy; working at 27.12 MHz). The holding time stablished for each equipment was 10.9 s (CH) and 9.08 s (RF). The freshly pasteurised salmorejo was cooled to 25 °C in a tubular heat exchanger and aseptically packaged in 250 mL translucent HDPE bottles with white polypropylene closures (Cat. No.342089; Nalguene narrow-mouth bottles; Thermo Fisher, Barcelona, Spain). For the shelf-life study, samples were kept at 4 °C in darkness for up to 5 months at one-month intervals. All samples were analysed in triplicate. A more detailed procedure can be consulted in a previous study [29].

### 2.3. Vitamin C

Vitamin C (L-ascorbic + dehydroascorbic acid) was determined by HPLC-DAD [31] using an Infinity II 1260 Series system (Agilent Technologies, Santa Clara, CA, USA). Detailed information concerning ascorbic acid analysis (sample pretreatment, HPLC column and operating conditions, chemical standard and calibration) is available in [29]. To quantify dehydroascorbic acid, a 1.5 mL sample extract was mixed with 1.5 mL 1,4-dithiothreitol 2% (*w*/*v*) water solution and kept in the dark for 2 h. This reaction converts dehydroascorbic acid to L-ascorbic acid. Sample analysis was then repeated to quantify dehydroascorbic acid and total vitamin C. Results were expressed as mg acid per 100 g salmorejo.

### 2.4. Vitamin E and Total Carotenoids

#### Obtention of Sample Extract

Extraction was conducted according to Sadler et al., (1990) [32] with some modifications. 0.5 g salmorejo was mixed with 10 mL of hexane:acetone:ethanol (2:1:1, *v*/*v*) and sonicated at room temperature for 5 min. The sample was homogenised with 2 mL distilled and kept at room temperature and in the dark for 15 min to facilitate phase separation. The non-polar phase was transferred to a round-bottomed distillation flask and evaporated in a Hei-vap rotavapor (Heidolph, Schwabach, Germany) at 35 °C and 270 HPa for 10 min. Dry residue was redissolved in 1 mL tert-butyl methyl ether:methanol (1:1, *v*/*v*), centrifuged at 6596× *g* for 12 min (D-37520 Biofuge Pico centrifuge, Heraeus, Germany) and filtered with a 0.45 µm Millipore filter to obtain the sample extract ready for injection into the chromatographic system.

### 2.5. Vitamin E

Next, 20 µL sample extracts were injected into an HPLC-DAD Infinity II 1260 Serie system with a Brisa LC2 C18 column (25 × 0.46 cm, with 5 µm pore size). Operating conditions were: (i) Flow rate: 1 mL min^−1^; (ii) Mobile phase: methanol in isocratic; (iii) Temperature: 27 °C; (iv) Analysis time duration: 20 min; and (v) Detection wavelength: 204 nm. α-tocopherol (Sigma, St. Louis, MO, USA) was used as standard. Then 0.01 g α-tocopherol (Sigma, St. Louis, MO, USA) were completed with a methanol: methyl tert-butyl ether (1:1; *v*/*v*) solution in 20 mL volumetric flask to prepare standard solution. A calibration line (y = 110.76x − 26.384; R^2^ = 0.9999) of the standard solution at concentrations from 50 to 500 µg α-tocopherol mL^−1^ was used for quantification. Results were expressed as mg α-tocopherol per 100 g salmorejo.

### 2.6. Carotenoids

Carotenoids were analysed according to Böhm (2001) [15], with some modifications. A 100 µL sample extract was injected into a 1200 HPLC-DAD system (Agilent Technologies, Barcelona, Spain) with a C30 column (25 × 0.46 cm, 5 µm pore size) (Análisis Vínicos, Villarobledo, Spain). Operating conditions were as follows: (i) Flow rate: 1 mL min^−1^. (ii) Mobile phase: tert-butyl methyl ether (A) and methanol (B), where the gradient started with 2% A in B to reach 35% A at 35 min, 60% A at 45 min, 60% at 55 min and returning to initial conditions (2% A in B) for 5 min prior to next injection. (iii) Temperature: 17 °C. (iv) Analysis time duration: 60 min. Ultraviolet spectra and retention times by chromatographic comparisons with authentic standards were used to identify carotenoids. β-carotene was quantified at 450 nm, E-lycopene and its Z-isomers were quantified at 472 nm. Standards of lycopene Z-isomers were unavailable, thus were identified based on their retention times and spectral characteristics described in the literature [25,33]. A calibration line 0.1 mg mL^−1^ (*w*/*v*) of β-carotene (y = 13.99x − 62.259; R^2^ = 0.9998) and E-lycopene (y = 15.094x + 88.165; R^2^ = 0.9998) (LGC Standards, Teddington, UK) at concentrations from 0.010 to 2.6 mg 100 g^−1^ (β-carotene) and 0.05 to 1.1 mg 100 g^−1^ (E-lycopene) were used for quantification. Results were expressed as mg carotenoids per 100 g salmorejo.

### 2.7. Antioxidant Capacity Assays

#### Total Phenolic Content (TPC)

The TPC was analysed according to a modified version of the Folin–Ciolcateu method [34]. A total of 5 g salmorejo was completed with methanol in a 10 mL volumetric flask under stirring and kept at room temperature and in the dark for 30 min. The sample was centrifuged at 4554× *g* and 4 °C for 10 min. The supernatant was filtered with 0.25 Millipore SAS filter (Molsheim, France) to obtain sample extract. A 250 µL sample extract was mixed under stirring with 5 mL distilled water and 800 µL Folin–Ciocalteu reagent (Sigma, St. Louis, MO, USA) into a 10 mL volumetric flask. After 8 min, 1.2 mL Na_2_CO_3_ 20% (*w*/*v*) water solution was added to the sample and the flask was completed with distilled water. The sample mixture was kept at 20 °C and in the dark for 2 h in a water bath. Sample absorbance was measured at 760 nm. The blank was determined by replacing the sample extract with distilled water. A calibration line (y = 0.1332x − 0.1946; R^2^ = 0.9994) of 5 mg mL^−1^ (*w*/*v*) Gallic acid (GA) (Merck KGaA, Darmstadt, Germany) water solution at concentrations from 0.1 to 5.5 µg GA mL^−1^ was used for quantification. Results were expressed as mg Gallic Acid Equivalent (GAE) per 100 g salmorejo.

### 2.8. Obtention of Sample Extract

In total, 2 g of salmorejo was completed with methanol in a 10 mL volumetric flask and kept under stirring at room temperature and in the dark for 10 min. The mix was centrifuged in a Digicen 21 R centrifuge (Orto Alresa, Madrid, Spain) at 3500× *g* at 4 °C for 10 min. The supernatant was collected to obtain sample methanolic extract and stored at −20 °C until further analysis.

#### 2.8.1. 2.2′-Azinobis-3-Ethylbenzothiazoline-6-Sulfonic (ABTS•) Radical Cation Decolouration Assay

The ABTS assay was conducted according to Re et al., (1999) [35]. To obtain reagent solution, 1 mL 2.45 mM K_2_S_2_O_8_ water solution was mixed with 10 mL of 7 mM ABTS water solution (Merck, Darmstadt, Germany) and kept at room temperature for 16 h. The absorbance at 734 nm of the reaction solution was adjusted to 0.7 using ABTS + K_2_S_2_O_8_ solution and distilled water to obtain the adjusted reaction solution. A UV-Vis Genesys™ 180 spectrophotometer (Thermo Fisher Scientific, Madison, WI, USA) was used. A 15 µL sample extract was mixed with 985 µL ABTS + K_2_S_2_O_8_ reagent solution and kept at room temperature and in the dark for 6 min before measuring absorbance at 734 nm. The blank was determined by replacing the sample extract with distilled water. A 10 mM ethanolic solution of Trolox (±-6-hydroxy-2,5,7,8-tetramethylchromane-2-carboxylic acid; Sigma, St. Louis, MO, USA) was used as standard. A calibration line (y = 111.72x + 0.0138; R^2^ = 0.9987) of Trolox Equivalents (TE) at concentrations from 5 to 500 µg mL^−1^ was used for quantification. Results were expressed as mg TE per 100 g salmorejo.

#### 2.8.2. 2,2-Diphenyl-1 Picrylhydrazyl (DPPH•) Radical-Scavenging Activity

The DPPH assay was conducted according to Brand-Williams et al., (1995) [36] with some modifications. To obtain reagent solution, a 0.9 mM DPPH methanolic solution was kept at room temperature and in the dark for 30 min and absorbance at 517 nm was adjusted to 1.0 using methanol. A 15 µL sample extract and 985 µL of DPPH solution was mixed and kept at room temperature and in the dark for 10 min. Sample absorbance was measured at 517 nm. The blank was determined by replacing the sample extract with distilled water. A calibration line (y = 83.729x + 0.0279; R^2^ = 0.9991) of 10 mM TE at concentrations from 10 to 500 µg TE mL^−1^ was used for quantification. Results were expressed as mg TE per 100 g salmorejo.

#### 2.8.3. Ferric Ion Reducing Antioxidant Power (FRAP)

The FRAP assay was conducted according to Benzie and Strain, (1999) [37] with some modifications. To obtain the FRAP reagent, a mixture of 300 mM acetate buffer (pH 3.6), 10 mM 2,4,6-Trypyridyl-s-Triazine (TPTZ) solution in 40 mM HCl and 20 mM ferric chloride (10:1:1; *v*/*v*/*v*) was freshly prepared. In total, 1.2 mL of FRAP reagent solution was mixed with 40 µL sample extract and 120 µL distilled water. Sample absorbance was measured at 593 nm. The blank was determined by replacing the sample extract with distilled water. A calibration line (y = 0.0003x + 0.0308; R^2^ = 0.9991) of 1 mM FeSO_4_·7 H_2_O (Panreac, Castellar del Vallès, Spain) at concentrations from 10 to 500 µM Fe^2+^ L^−1^. Results were expressed as µM Fe^2+^ per 100 g salmorejo.

### 2.9. Statistical Analysis

A randomised factorial design was performed for the experiment. For the first trial, treatments were heating method (CH vs. RF) and pasteurisation temperature (70–100 °C at 5 °C intervals). Sample size was *n* = 144 (3 manufacturing batches × 3 bottled samples × 2 heating methods × 8 temperatures, including raw samples). For the second trial, treatments were heating method (CH or RF, at 80 °C) and storage time (0–5 months, at one-month intervals). Sample size was *n* = 126 (3 manufacturing batches × 3 bottled samples × 2 heating methods × 7 retailing times, including raw samples). The effects of treatments on dependent variables were determined by two-way ANOVA (Tukey range test; *p* < 0.05). Data were analysed with the Statistix 8.0 software for Windows (Analytical Software, Tallahassee, FL, USA).

## 3. Results

### 3.1. Effects of Heating Method and Pasteurisation Temperature

Antioxidants quantified in the salmorejo pasteurised upon different conditions are shown in Table 1. CH and RF treatments provided a product with similar levels of antioxidants, with some exceptions. The highest concentrations of L-ascorbic acid, dehydroascorbic acid and total vitamin C corresponded to raw salmorejo (more than 12, 8 and 20 mg 100 g^−1^, respectively). In the pasteurised product, L-ascorbic acid level strongly decreased at 70 °C (over 9 mg 100 g^−1^), was higher at 75–90 °C (over 11 mg 100 g^−1^) and decreased again at 95–100 °C (over 8 mg 100 g^−1^). Dehydroascorbic acid content tended to increase with temperature in CH (from 6.0 to 7.8 mg 100 g^−1^) and RF (from 5.6 to 8.2 mg 100 g^−1^) samples. The highest contents of total vitamin C were recorded at 80–95 °C (over 18 mg 100 g^−1^) in CH and RF samples. Unlike those seen for vitamin C, raw and pasteurised salmorejo had similar levels of α-tocopherol (over 1.0 mg 100 g^−1^). Again, the lowest levels of α-tocopherol corresponded to the CH and RF samples pasteurised at 70 °C (over 0.84 mg 100 g^−1^). α-tocopherol level tended to increase with temperature, although there were some fluctuations at intermediate temperatures and effects were not too clear. The main carotenoids, in order of abundance, were E-lycopene, β-carotene, 15-*cis*-isomer of lycopene and 9-*cis*-isomer of lycopene. Other tomato carotenoids were below the limit of quantification (LOQ) of the method. That was also the case for lutein (LOQ = 0.004 mg 100 g^−1^) and α-carotene (LOQ = 0.013 mg 100 g^−1^). The lowest contents of total carotenoids (<1 mg 100 g^−1^) again corresponded to the CH and RF samples at 70 °C. The total content of all-*trans* + *cis* Lycopene tended to increase with temperature in CH (from 1.0 to 2.2 mg 100 g^−1^) and RF (from 1.0 to 2.2 mg 100 g^−1^) samples. β-carotene content also tended to increase with temperature in CH (from 0.25 to 3.8 mg 100 g^−1^) and RF (from 0.25 to 3.5 mg 100 g^−1^) samples. The total content of carotenoids tended to increase with temperature in CH (from 0.6 to 2.3 mg 100 g^−1^) and RF (from 0.7 to 1.8 mg 100 g^−1^) samples, although, as seen for α-tocopherol, there were also fluctuations at intermediate temperatures. Regardless of the heating method used, pasteurisation at low temperature (70 °C) resulted in a salmorejo with a lesser antioxidant content. Increasing the temperature up to 100 °C led to some decrease in vitamin C content but not in the respective contents of α-tocopherol and carotenoids, which may even be increased.

AC values recorded in salmorejo pasteurised upon different conditions are shown in Table 2. The lowest TPC corresponded to samples pasteurised at 70 °C (over 27 mg GAE 100 mL^−1^). TPC tended to increase with temperature in CH (from 28 to 31 mg GAE 100 mL^−1^) and RF (from 27 to 31 mg GAE 100 mL^−1^) samples, reaching at 100 °C similar contents than those recorded in raw product (over 32 mg GAE/100 mL^−1^). The DPPH values of raw product (9–11 mg GAE 100 g^−1^) also decreased with CH or RF pasteurisation. The lowest DPPH values corresponded to samples pasteurised at 70 °C (6–7 mg GAE 100 g^−1^) and 95–100 °C (7–8 mg TE 100 g^−1^). RF samples had higher DPPH values than CH samples at various temperatures. Pasteurisation effects on ABTS values were less clear, with minor differences among raw (16–17 mg TE 100 g^−1^) and pasteurised (16–19 mg TE 100 g^−1^) samples processed with CH or RF. The lowest FRAP values again corresponded to samples pasteurised at 70 °C (196–202 mg Fe^2+^ 100 mL^−1^). FRAP values tended to increase with temperature in CH (from 202 to 237 Fe^2+^ 100 mL^−1^) and RF (from 204 to 228 Fe^2+^ 100 mL^−1^) samples. Regardless of the heating method, the AC improved when salmorejo was pasteurised at temperatures higher than 70 °C.

### 3.2. Changes in Antioxidant Properties during Chill Storage

Changes over time in antioxidant quantification made in pasteurised (at 80 °C) salmorejo are shown in Table 3. Again, results obtained for CH and RF salmorejo were similar. The content of L-ascorbic acid strongly decreased from 19–21 to less than 1.2 mg 100 g^−1^ by the first month and then remained over 1 mg 100 g^−1^ during the rest of storage. The decrease over time in the content of dehydroascorbic acid was less pronounced in CH (from 10.2 to 3.1 mg 100 g^−1^) and RF (from 8.5 to 3.7 mg 100 g^−1^) samples at the first month, showing values less than 1.1 mg 100 g^−1^ from the second month onwards. Total vitamin C content similarly decreased in CH (from 29.5 to 4.3 100 g^−1^) and RF (from 29.5 to 4.8 mg 100 g^−1^) samples at the first month and then remained low (<2 mg 100 g^−1^) in both samples during the rest of storage. Unlike those seen for vitamin C, salmorejo retained most of its α-tocopherol during its shelf life. α-tocopherol content remained over 1.2 mg 100 g^−1^ throughout chill storage in the CH and RF salmorejo at 80 °C. In contrast, there were changes in carotenoid content during chill storage. After five months, E-Lycopene content tended to decrease in CH (from 1.6 to 1.0 mg 100 g^−1^) and RF (from 1.5 to 0.9 mg 100 g^−1^) samples; 15-*Cis*-Isomer of Lycopene content was not affected by storage time, there was some formation of 9-*Cis*-Isomer of Lycopene during storage, while β-Carotene content decreased from 0.35 mg 100 g^−1^ at the beginning of storage to 0.25 mg 100 g^−1^ after five months. Total carotenoid content decreased in CH and RF samples (from 2.1 to 1.3 mg 100 g^−1^) after five months. Chill storage had a detrimental effect on the levels of salmorejo carotenoids, although there were fluctuations over time.

Changes over time in the AC of pasteurised (at 80 °C) salmorejo are shown in Table 4. AC values were similar in CH and RF salmorejo, except for the DPPH value at the end of storage (lower in RF). TPC tended to decrease with chill storage (from 36 to 32 mg GAE 100 mL^−1^), so that pasteurised-chilled salmorejo kept most of its phenolic antioxidant capacity during shelf life. DPPH value also decreased from 21.7 to 7.7 mg TE 100 g^−1^ after five months of storage, meaning that the product gradually lost most of its antiradical scavenging activity to DPPH. ABTS values decreased more in RF (from 22 to 13 mg TE 100 g^−1^) than in CH (from 22 to 8 mg TE 100 g^−1^) stored product; in this case, the loss of most antiradical scavenging activity to ABTS was recorded at the first month. FRAP values also decreased with chill storage in CH and RF samples (from 230 to 160 mg Fe^2+^ 100 mL^−1^). AC decreased over time in pasteurised-chilled salmorejo, particularly at the beginning of storage. The four assays allowed a good discrimination among samples with different shelf lifetime.

## 4. Discussion

Salmorejo is a tomato-oil emulsion containing hydrophilic (e.g., L-ascorbic and dehydroascorbic acids) and lipophilic (e.g., α-tocopherol and carotenoids) antioxidants involved in different protection mechanisms during processing and further storage. Both heating technologies, conventional or RF, provided salmorejo with similar levels of antioxidants and AC values, though there were some minor differences concerning levels of L-ascorbic acid, a thermally unstable compound. In salmorejo, conversion of L-ascorbic to dehydroascorbic acid—an early oxidation index—began before pasteurisation and was probably favoured by the grinding and homogenisation treatments conducted in aerobic equipment. Heating method had no clear effect on retention of vitamin C, whose total contents were similar for CH and RF salmorejo at most tested temperatures. Retention of α-tocopherol or carotenoids was quite similar in the salmorejo pasteurised by both technologies. These results were expected because flow conditions and holding times were adjusted in both types of continuous pasteurisers to reach similar levels of microbiological inactivation (Z-values) [29]. Dielectric heating has been barely studied in tomato-oil emulsions with salt, a dielectric component involved in heat generation; at RF frequencies, the dielectric constant decreases with increasing temperature in samples without salt, while this tendency was reversed in samples with added salt [38]. There are some available data on tomato products pasteurised by conventional or dielectric heating. In tomato puree samples located in a water bath, heating by RF (27.12 MHz) from 25 to 90 °C (core temperature) allowed a good retention of L-ascorbic acid [7]. Arjmandi et al., (2018) [22] studied retention levels for antioxidants and AC in a tomato puree continuously pasteurised by CH (96 °C for 35 s) or MW (combinations at 390–3150 W for 150–848 s). According to these authors, microwaved puree retains more vitamin C and shows more AC than the conventional product, particularly when high-power/short-time microwaving is applied. In another study, the AC of a pasteurised tomato juice (85 °C for 5 min) also improved with MW heating [39]. Both studies show that MW improves heat penetration and favours tomato cell-wall disruption and lycopene extraction with an improvement in AC. In the present study, salmorejo was pasteurised upon milder conditions (HTST) and it is unlikely that heating technology can produce different thermal effects on the product.

**Figure 1:**
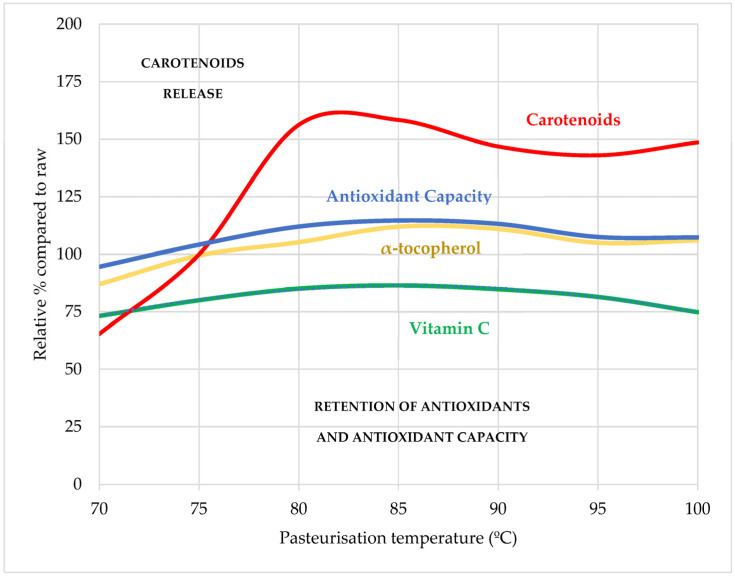
Effects of pasteurisation temperature on the relative changes in antioxidant content and antioxidant capacity of salmorejo. All values expressed as average (CH + RF) relative percentages respect to the raw product (100%). Antioxidant capacity: average relative values for TPC, DPPH, ABTS and FRAP assays.

**Figure 2:**
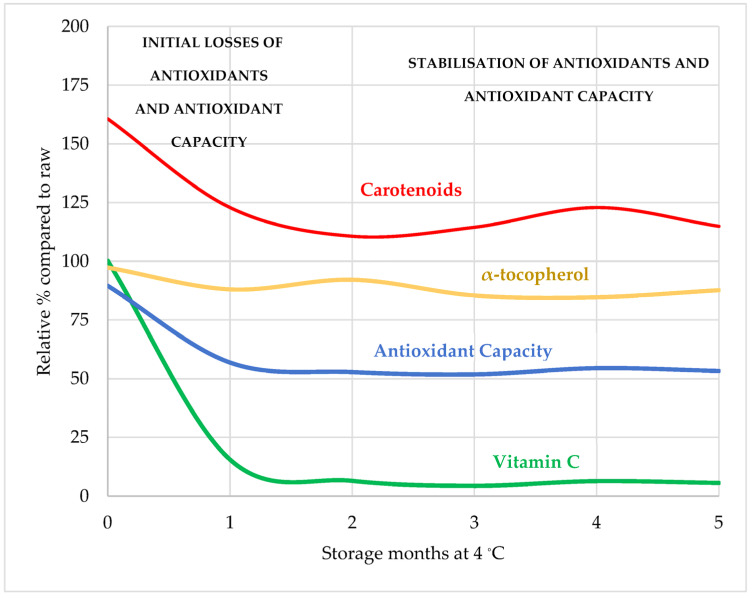
Effects of storage time on the relative changes in antioxidant content and antioxidant capacity of pasteurised-chilled salmorejo. All values expressed as average (CH + RF) relative percentages respect to the raw product (100%). Antioxidant capacity: average relative values for TPC, DPPH, ABTS and FRAP assays.

The effects of pasteurisation temperature and shelf-life time on the antioxidant properties of salmorejo are illustrated in Figure 1 and Figure 2. Vitamin C, the most abundant antioxidant of the three studied, appears solubilised in the aqueous phase of this emulsion, which accounts for about 95% of its composition. In the pasteurisation trial, salmorejo lost up to a third of this vitamin C (20 mg 100 g^−1^) depending on the temperature applied. Retention of vitamin C was superior in the product pasteurised at 80–90 °C, suggesting that oxidation reactions leading to the degradation of this vitamin were probably attenuated at intermediate temperatures. The shelf-life trial confirmed that the salmorejo pasteurised at 80 °C retains practically all its vitamin C (29–30 mg 100 g^−1^). However, most of its vitamin C degraded after one month of chill storage. Therefore, pasteurisation treatment appears to be less relevant than the storage time at the moment of consumption for the retention of vitamin C in this product. Different studies agree that most vitamin C is retained by mild heated tomato products, including salmorejo [40], gazpacho (a similar tomato-oil soup with fresh pepper and fresh onion) [41], purees [22,23,24] and vegetable beverages [21]. At the beginning of shelf-life, salmorejo contained higher levels of vitamin C than those reported for the respective commercial versions of pasteurised salmorejo (7.9 mg 100 g^−1^) [40] and pasteurised gazpacho (9.0–11.4 mg 100 mL^−1^) [26,28,41], being within the range (9–36 mg 100 g^−1^) reported for pasteurised tomato purees and smoothies [22,23]. Other shelf-life studies reported losses of vitamin C in similar products. In gazpacho stored for 30 days, vitamin C degraded to a greater extent when the product was pasteurised for 5 min at 60 °C (from 20 to 13 mg 100 g^−1^) than at 90 °C (from 18 to 15 mg 100 g^−1^) [42] while, in crushed tomato, vitamin C decreased from 8 to 3 mg 100 g^−1^ after 180 days at 10 °C [43]. Salmorejo lost most of this vitamin C at the first month of shelf-life coinciding with the time period of higher activities of PPO and POD enzymes [30].

Thermal effects on tomato carotenoids were less clear because both degradation and overextraction can occur. As for vitamin C, the level of carotenoids was lower at 70 °C, showing values even below those found for raw salmorejo, probably due to oxidase residual activities [30]. By contrast, carotenoid content increased at temperatures higher than 70 °C. This would be due to homogenising and heating treatments being able to disrupt cell membranes and carotenoid-protein complex, making carotenoids more accessible for extraction [44]. This fact has also been checked for carotenoids and tocopherols in oven-baked tomatoes [12]. Quantified carotenoids in the shelf-life trial were almost double in the freshly pasteurised than in the raw salmorejo. Once released, carotenoids moderately degraded during the first month of shelf life and then remained stable, which is a positive nutritional trait. Several studies agree that HTST tomato products retain most of their carotenoids intact [22,23,24,40]. Intense thermal treatments (e.g., cooking) are required for tomato carotenoids to be degraded. Lycopene is stable in tomato products heated at 80–100 °C, while its isomerisation increased at 120–140 °C, resulting in a degradation of total lycopene and cis-isomers [18]. Lycopene degrades following a first order kinetic in the tomato juice heated at 80 to 100 °C for 20 to 80 min [19]. In crushed tomato, lycopene content (3.5 mg 100 g^−1^) decreased by up to 55% after heating at 100 °C for 120 min [17]. Thermal stability can be different for carotene or lycopene. In tomato juice, pasteurisation (at 90 °C for 90 s) reduced β-carotene but not lycopene content, as the former is more susceptible to thermal degradation or isomerisation [16]. At the beginning of shelf life, the salmorejo pasteurised at 80 °C contained less lycopene and similar levels of β-carotene than those values reported for a pull of commercial salmorejo (0.67 and 0.31 mg 100 g^−1^, respectively) [40]. Salmorejo was also poorer in lycopene and β-carotene than several commercial gazpachos (1.6–4.8 and 1.9–5.1 mg 100 g^−1^, respectively) [28], or than tomato puree (6.5–7.7 and 10–14 mg 100 g^−1^, respectively) [23]. Salmorejo contains tomato skin rich in carotenoids [8] but is prepared with other ingredients different to tomatoes. Other studies agree that carotenoids remain quite stable during the shelf life of tomato products. In crushed tomato, lycopene content decreased from 15 to 14 mg 100 g^−1^ after 180 days of storage at 10 °C [43], while decreases in carotenoid content of up to 7% (*cis* and *trans* lycopene) and to 19% (α and β carotene) were found in commercial gazpachos kept at 3 °C until 12 weeks [3].

As seen for vitamin C or carotenoids, retention of α-tocopherol was clearly lower in the salmorejo heated at 70 °C, perhaps since its degradation was favoured by oxidase residual activities [30], while, in contrast, there was no clear degradation of this antioxidant at higher temperatures. Most salmorejo α-tocopherol probably came from the EVOO used as an ingredient. EVOO is exclusively obtained by mechanical procedures, being more stable to oxidation than other edible oils [45]. Tocopherol stability in tomato-oil emulsions might depend on chemical interactions with other compounds that can produce its degradation or regeneration. Salmorejo is not particularly prone to lipid oxidation, though it can contain oxidised lipids at low concentrations (<0.5 mg malondialdehyde kg^−1^) just after pasteurisation and during shelf life [29,30]. α-tocopherol perhaps contributed toward stabilising lipids and maintaining constant levels during shelf life, which suggests that it was regenerated by other antioxidants. This great stability of α-tocopherol during the long shelf life of salmorejo of storage can be considered another positive nutritional aspect. The α-tocopherol content recorded in salmorejo (over 1 mg 100 g^−1^ at 80 °C) was coherent with those published for a nutritional estimation of salmorejo (1.3 mg 100 g^−1^) containing 10.8 g olive oil 100 g^−1^ product [46].

Retention of antioxidants by salmorejo can also be explained by factors such as their concentrations, specific chemical activities, synergisms with other antioxidants and their susceptibility to enzymes. Vitamin C species are easily oxidizable molecules involved in many oxidation-reduction reactions. L-ascorbic acid does not interact directly with PPO but prevents browning by reducing oxidised substrates, being frequently added as an antioxidant agent to prevent enzyme-catalysed browning reactions [47]. L-ascorbic acid can act as an electron donor in the reaction of the ascorbate–glutathione cycle where an ascorbate peroxidase enzyme can reduce H_2_O_2_, the main by-product of POD activity, and as a direct ROS scavenger, and can protect both carotenoids and tocopherols against oxidation [48]. A study made in vitro with liposomes revealed that, depending on oxygen pressure, β-carotene can be a less potent ROS scavenger against lipid peroxidation than α-tocopherol; in this study, L-ascorbic acid showed some synergism with α-tocopherol but not with β-carotene. On the other hand, thermal behaviour of tomato carotenoids can be different. Lycopene tends to form crystals inside plant cells that confer a great thermal stability, while β-carotene is more sensitive to heat applied for tomato processing [44]. Furthermore, heat can induce isomerisation of the all-*trans* to *cis* lycopene forms, which increase with temperature and processing time [49]. This may explain why the 15-*cis*- and 9-*cis*-isomers of lycopene were more abundant in heated rather than in raw salmorejo. Tomato-based product such as gazpacho contain phenolic antioxidants (e.g., hydroxycinnamic acids and flavonoids) able to stabilise the carotenoids [3]. Lycopene stability during the shelf life of tomato heated products is favoured by factors such as enzyme inactivation, the presence of tomato skin and the protection of other antioxidants (e.g., β-carotene or α-tocopherol) [43]. The formation of O-quinones mediated by PPO enzyme brings accumulation of H_2_O_2_, which in turn causes the browning of polyphenols mediated by POD [50]. β-carotene content can decrease in tomato products depending on whether hydrolysis from linked proteins predominates over other oxidative reactions [43]. α-tocopherol is another ROS scavenger that inhibits the formation of oxidised secondary lipids from hydroperoxides. In salmorejo, α-tocopherol mainly appears solubilised with EVOO, rich in triglycerides, which facilitates its antioxidant activities on lipids. α-tocopherol stability depends on olive oil composition (free fatty acids, saturated and unsaturated fat ratio, phenols, etc.) and processing conditions (packing material, occluded oxygen, temperature, and lighting) [51]. Phenolic compounds (e.g., oleuropein and hydroxytyrosol) also present in EVOO can stabilise the α-tocopherol during heating [52]. EVOO tocopherols are prone to oxidative degradation during storage, being more affected by lighting than by room temperature [53].

Vitamins C and E and carotenoids likely contributed to the AC in salmorejo. In the pasteurisation trial, the lower AC assessed by the four assays (TPC, ABTS, ABTS and FRAP) corresponded to samples pasteurised at low temperatures (70 or 75 °C). AC provided by total phenols rose with increasing pasteurisation temperature, as occurred with carotenoid levels. Something similar occurred for ROS scavenging activities to ABTS and FRAP, while the highest ROS scavenging activity to DPPH was recorded at intermediate temperatures. These results can be explained by the different chemical nature of each assay [54]. The AC values obtained with water-methanolic sample extracts largely exceeds those obtained with organic solvents in these tomato-oil emulsions [26,28]. The Folin–Ciolcateu reaction measures the antioxidant response of phenolic antioxidants, though other sample components (e.g., reducing sugars) can also show positive response in this reaction. The ABTS assay measures the ability of sample antioxidants to extinguish the ABTS• radical cation in lipophilic and hydrophilic environments. The DPPH assay measures the ability of antioxidants or other ROS to react with the DPPH• radical and is widely used for plant antioxidants. The FRAP is a redox assay that measures the antioxidant potential through reduction of ferric iron (Fe^3+^) to ferrous iron (Fe^2+^) by sample antioxidants, being useful in determining the AC of water-soluble phenols. In salmorejo, intensifying pasteurisation treatment does not necessarily entail a loss of AC because carotenoid release by heating likely also contributes to antioxidant status. In contrast, chill storage has a detrimental effect on antioxidant status in this product. During shelf life, AC, vitamin C and carotenoid contents decreased over time. This precedes the appearance of objective oxidation signs (discolouration, stress thinning or lipid oxidation) in salmorejo [30]. Several authors agree that enzyme deterioration negatively contributes to AC in pasteurised-chilled tomato-oil emulsions [42,55]. AC of fruit and vegetable products can also be negatively affected by non-enzymatic oxidation, including browning caused by Maillard reaction products [56]. TPC (around 30 mg GAE 100 mL^−1^) and DPPH values (around 9 mg TE 100 mL^−1^) of salmorejo pasteurised at 80 °C were coherent with those values reported for a pull of commercial salmorejo (27 mg and 13 mg TE 100 g^−1^) [40]. Vallverdú-Queralt et al. (2012) [3] reported slight losses of AC (DPPH and ABTS) after 12 weeks of chill storage in commercial pasteurised gazpachos. In sterilised tomato pulp, an increase over time (180 days at 20 °C) in TPC from 9.4 to 12.5 GAE 100 g^−1^ corresponded with slight increases in content of lycopene (from 14.0 to 14.5 mg 100 g^−1^) and β-carotene (from 0.7 to 1.1 mg 100 g^−1^), while vitamin C content decreased (from 8.6 to 4.2 mg 100 g^−1^) [43]. This suggests that carotenoids can be released or regenerated during the shelf life of heated tomato, thus increasing AC. A problem with the AC values obtained from different food samples and analytical procedures is that information is not often comparable. Moreover, HTST treatments can modify carotenoids content in tomato products, affecting the results of AC. A study on AC (TE 100 g^−1^) made in gazpacho [26] found that this product shows more hydrophilic (18.9) than lipophilic (9.1) AC (TE 100 g^−1^); the AC measured was higher for L-ascorbic acid (9.1) than for lycopene (0.5) or β-carotene (0.6) at the same concentrations present in the sample [26]. Similar findings have been reported for total ROS scavenging activity in commercial gazpachos with different pasteurisation treatments [28] and in tomato juices, canned tomatoes and tomato paste [27].

## 5. Conclusions

Salmorejo is a good dietary source of vitamin C (L-ascorbic and dehydroascorbic acids), vitamin E (α-tocopherol) and carotenoids (lycopene and β-carotene) from tomato and olive oil. Vitamin C is more abundant but less resistant to heating and storage than carotenoids and α-tocopherol. HTST pasteurisation allows a good retention of these antioxidants. Either conventional heat exchangers or radiofrequency equipment can be used with similar results when operating conditions are adjusted to standardise thermal effects. Pasteurisation temperature in the studied range (70–100 °C) may produce some detriment in salmorejo antioxidants. Using excessively low temperatures (70 °C) may lead to partial losses of the three antioxidants, probably due to oxidase residual activities. Intensifying the thermal treatment improves this issue with minor losses of the thermolabile vitamin C and increased carotenoid content, thus contributing to antioxidant stability in this product. Using a suitable temperature (80 °C) does not prevent most vitamin C from being degraded at the first month, while α-tocopherol, and, to a lesser extent, carotenoids, show good retention levels during shelf life under refrigeration. Vitamins C and E and carotenoids, either by degradation, regeneration or releasing, likely contribute to the AC in salmorejo. Phenol antioxidant activity (TPC), ROS scavenging activities (ABTS and DPPH) and redox potential (FRAP) values confirmed this finding. The pasteurised-chilled salmorejo shows good antioxidant properties with potential health implications, a positive nutritional aspect for the consumers of this tomato-oil homogenates. The losses of antioxidants and AC due to pasteurization would be of little relevance compared to the losses accumulated during shelf life.

## Figures and Tables

**Table 1:** Concentrations (mg 100 g^−1^) of vitamin C, α-tocopherol and carotenoids in salmorejo pasteurised at different temperatures by conventional (CH) or radiofrequency (RF) continuous heating.

Temperature (°C)		Raw	70	75	80	85	90	95	100	RMSE
		M	M	M	M	M	M	M	M	
L-ascorbic acid	CH	12.0 ^a^	8.98 ^i^	10.5 ^de^	11.1 ^cd^	11.5 ^bc^	9.90 ^g^	9.16 ^j^	8.58 ^j^	0.117
	RF	12.5 ^a^	9.55 ^h^	11.0 ^cd^	10.2 ^ef^	10.7 ^de^	11.7 ^ab^	9.50 ^h^	7.33 ^k^	
Dehydroascorbic acid	CH	8.09 ^a^	6.04 ^cd^	6.03 ^cd^	6.38 ^bc^	6.10 ^cd^	6.40 ^bc^	6.74 ^ab^	7.22 ^ab^	0.371
	RF	8.64 ^a^	5.60 ^e^	7.03 ^ab^	7.43 ^ab^	7.41 ^ab^	7.00 ^ab^	8.16 ^a^	7.77 ^ab^	
Total vitamin C	CH	20.1 ^a^	15.0 ^f^	16.5 ^cd^	17.4 ^bc^	17.5 ^bc^	16.4 ^de^	15.9 ^e^	15.8 ^e^	0.343
	RF	21.2 ^a^	15.2 ^f^	16.6 ^cd^	17.6 ^bc^	18.1 ^bc^	18.6 ^b^	17.7 ^bc^	15.1 ^f^	
α-tocopherol	CH	0.92 ^cd^	0.83 ^d^	0.96 ^cd^	1.04 ^bc^	1.14 ^ab^	1.19 ^a^	1.08 ^ab^	1.08 ^ab^	0.344
	RF	0.99 ^bc^	0.84 ^d^	0.95 ^cd^	0.98 ^bc^	1.05 ^ab^	0.97 ^cd^	0.98 ^bc^	1.00 ^bc^	
E-Lycopene	CH	1.25 ^f^	0.62 ^h^	1.16 ^fg^	2.06 ^a^	1.90 ^ab^	2.11 ^a^	1.54 ^cd^	2.03 ^a^	0.698
	RF	0.93 ^hg^	0.69 ^h^	1.01 ^g^	1.44 ^de^	1.74 ^bc^	1.19 ^fg^	1.34 ^def^	1.26 ^efg^	
15-*Cis*-Isomer of Lycopene	CH	0.03 ^d^	0.03 ^d^	0.04 ^d^	0.12 ^a^	0.07 ^bc^	0.12 ^a^	0.08 ^c^	0.08 ^c^	0.067
	RF	0.04 ^d^	0.03 ^d^	0.04 ^d^	0.11 ^ab^	0.09 ^bc^	0.04 ^d^	0.08 ^c^	0.07 ^c^	
9-*Cis*-Isomer of Lycopene	CH	0.00 ^d^	0.00 ^d^	0.01 ^cd^	0.03 ^ab^	0.02 ^bc^	0.04 ^a^	0.04 ^a^	0.04 ^a^	0.034
	RF	0.00 ^d^	0.00 ^d^	0.00 ^d^	0.02 ^bc^	0.02 ^bc^	0.01 ^cd^	0.03 ^ab^	0.02 ^bc^	
All-*Trans* + *cis* Lycopene	CH	1.28 ^f^	0.64 ^h^	1.21 ^fg^	2.23 ^a^	2.00 ^ab^	2.27 ^a^	1.65 ^cd^	2.16 ^a^	0.735
	RF	1.00 ^hg^	0.72 ^h^	1.05 ^g^	1.56 ^de^	1.85 ^bc^	1.24 ^fg^	1.45 ^def^	1.35 ^efg^	
β-Carotene	CH	0.31 ^bc^	0.25 ^c^	0.33 ^b^	0.34 ^b^	0.35 ^ab^	0.36 ^ab^	0.33 ^b^	0.38 ^a^	0.123
	RF	0.30 ^bc^	0.25 ^c^	0.27 ^c^	0.34 ^b^	0.35 ^ab^	0.33 ^b^	0.37 ^ab^	0.35 ^ab^	
Total carotenoids	CH	1.60 ^ef^	0.90 ^g^	1.54 ^ef^	2.57 ^a^	2.34 ^ab^	2.63 ^a^	1.98 ^cd^	2.54 ^a^	0.792
	RF	1.26 ^f^	0.97 ^g^	1.32 ^f^	1.90 ^cd^	2.19 ^bc^	1.57 ^ef^	1.81 ^de^	1.71 ^de^	

Abbreviations: M: mean; RMSE: Root Mean Standard Error; ^a–k^ Means with different superscripts are different for *p* < 0.05 (Two-way ANOVA; Tukey Test).

**Table 2:** Antioxidant capacity measured in salmorejo pasteurised at different temperatures by conventional (CH) or radiofrequency (RF) continuous heating.

Temperature °C		Raw	70	75	80	85	90	95	100	RMSE
		M	M	M	M	M	M	M	M	
TPC	CH	32.6 ^a^	27.8 ^e^	28.3 ^cd^	30.7 ^b^	30.8 ^ab^	30.8 ^ab^	31.0 ^ab^	31.1 ^ab^	0.204
	RF	31.7 ^a^	27.3 ^e^	29.6 ^d^	30.4 ^b^	30.9 ^ab^	31.1 ^a^	31.7 ^a^	31.5 ^a^	
DPPH	CH	9.04 ^ab^	6.37 ^e^	8.64 ^bc^	9.80 ^ab^	10.5 ^a^	8.26 ^c^	7.57 ^cd^	7.77 ^cd^	0.399
	RF	11.2 ^a^	7.16 ^d^	8.56 ^bc^	8.62 ^bc^	9.45 ^ab^	10.9 ^a^	7.50 ^cd^	7.70 ^cd^	
ABTS	CH	15.8 ^b^	16.1 ^b^	16.0 ^b^	19.1 ^a^	17.6 ^ab^	17.7 ^ab^	18.5 ^ab^	18.5 ^ab^	0.716
	RF	17.6 ^ab^	16.0 ^b^	15.9 ^b^	17.5 ^ab^	17.3 ^ab^	17.1 ^ab^	18.0 ^ab^	16.9 ^ab^	
FRAP	CH	207.0 ^d^	202.4 ^d^	225.3 ^c^	226.6 ^bc^	233.9 ^ab^	232.2 ^ab^	234.0 ^ab^	237.4 ^a^	2.580
	RF	205.3 ^cd^	196.1 ^d^	204.3 ^cd^	216.7 ^cd^	223.7 ^c^	224.9 ^c^	227.3 ^bc^	222.8 ^c^	

Abbreviations: M: mean; RMSE: Root Mean Standard Error; TPC: Total Phenolic Content (mg Gallic acid 100 mL^−1^); DPPH: 2,2-diphenyl-1 picrylhydrazyl radical-scavenging activity (mg Trolox 100 g^−1^); ABTS: 2.2′-Azinobis-3-Ethylbenzothiazoline-6-Sulfonicradical Cation Decolouration Assay (mg Trolox 100 g^−1^); FRAP: Ferric Reducing Antioxidant Power Assay (mg Fe^2+^/100 mL^−1^); ^a–e^ Means with different superscripts are different for *p* < 0.05 (Two-way ANOVA; Tukey Test).

**Table 3:** Concentrations (mg 100 g^−1^) of vitamin C, α-tocopherol and carotenoids in salmorejo pasteurised at 80 °C by conventional (CH) or radiofrequency (RF) continuous heating and refrigerated for up to 5 months.

Storage Time (Months)		Raw	0	1	2	3	4	5	RMSE
		M	M	M	M	M	M	M	
L-ascorbic acid	CH	21.3 ^a^	19.2 ^b^	1.21 ^c^	1.17 ^c^	1.13 ^c^	1.06 ^c^	0.92 ^c^	0.085
	RF	21.3 ^a^	21.1 ^a^	1.10 ^c^	1.15 ^c^	1.01 ^c^	1.08 ^c^	0.88 ^c^	
Dehydroascorbic acid	CH	8.50 ^b^	10.2 ^a^	3.13 ^c^	1.02 ^d^	0.15 ^d^	0.78 ^d^	0.74 ^d^	0.286
	RF	7.88 ^b^	8.47 ^b^	3.74 ^c^	0.49 ^d^	0.30 ^d^	0.83 ^d^	0.74 ^d^	
Total vitamin C	CH	29.9 ^a^	29.5 ^a^	4.33 ^b^	2.18 ^c^	1.27 ^c^	1.83 ^c^	1.66 ^c^	0.277
	RF	29.0 ^a^	29.5 ^a^	4.84 ^b^	1.63 ^c^	1.31 ^c^	1.90 ^c^	1.63 ^c^	
α-tocopherol	CH	1.34 ^a^	1.34 ^a^	1.19 ^ab^	1.24 ^ab^	1.17 ^ab^	1.13 ^b^	1.17 ^ab^	0.032
	RF	1.34 ^a^	1.27 ^ab^	1.17 ^ab^	1.23 ^ab^	1.12 ^b^	1.14 ^b^	1.18 ^ab^	
E-Lycopene	CH	0.91 ^bc^	1.64 ^a^	0.97 ^bc^	0.96 ^bc^	0.99 ^bc^	1.11 ^bc^	1.09 ^bc^	0.073
	RF	0.86 ^c^	1.53 ^a^	1.32 ^ab^	1.04 ^bc^	0.86 ^c^	1.20 ^b^	1.07 ^bc^	
15-*Cis*-Isomer of Lycopene	CH	0.09	0.13	0.10	0.09	0.09	0.10	0.09	0.011
	RF	0.08	0.13	0.08	0.08	0.08	0.09	0.09	
9-*Cis*-Isomer of Lycopene	CH	0.01 ^c^	0.04 ^bc^	0.08 ^a^	0.08 ^a^	0.11 ^a^	0.09	0.09 ^a^	0.005
	RF	0.00 ^c^	0.04 ^bc^	0.04 ^bc^	0.06 ^b^	0.08 ^a^	0.08 ^a^	0.08 ^a^	
All-*Trans* + *cis* Lycopene	CH	1.01 ^c^	1.81 ^a^	1.15 ^bc^	1.13 ^bc^	1.43 ^ab^	1.29 ^bc^	1.27 ^bc^	0.051
	RF	0.93 ^c^	1.70 ^a^	1.43 ^ab^	1.19 ^bc^	1.00 ^c^	1.38 ^bc^	1.23 ^bc^	
β-Carotene	CH	0.35 ^a^	0.35 ^a^	0.33 ^ab^	0.29 ^bc^	0.28 ^bc^	0.27 ^bc^	0.26 ^c^	0.006
	RF	0.32 ^ab^	0.34 ^a^	0.31 ^ab^	0.29 ^bc^	0.27 ^bc^	0.28 ^bc^	0.25 ^c^	
Total carotenoids	CH	1.36 ^bc^	2.17 ^a^	1.47 ^bc^	1.42 ^bc^	1.71 ^ab^	1.57 ^bc^	1.53 ^bc^	0.066
	RF	1.25 ^c^	2.04 ^a^	1.75 ^ab^	1.48 ^bc^	1.29 ^bc^	1.65 ^ab^	1.48 ^bc^	

Abbreviations: M: Mean; RMSE: Root Mean Standard Error; ^a–d^ Means with different superscripts are different for *p* < 0.05 (Two-way ANOVA; Tukey Test).

**Table 4:** Antioxidant capacity measured in salmorejo pasteurised at 80 °C by conventional (CH) or radiofrequency (RF) continuous heating and refrigerated for up to 5 months.

Temperature °C		Raw	0	1	2	3	4	5	RMSE
		M	M	M	M	M	M	M	
TPC	CH	35.7 ^a^	36.3 ^a^	36.2 ^a^	34.6 ^b^	33.7 ^c^	32.4 ^d^	32.0 ^d^	0.174
	RF	36.4 ^a^	36.7 ^a^	35.1 ^b^	34.4 ^bc^	33.4 ^c^	32.0 ^d^	31.5 ^d^	
DPPH	CH	20.8 ^a^	21.7 ^a^	18.3 ^b^	14.7 ^c^	14.1 ^cd^	13.2 ^cd^	12.8 ^d^	0.293
	RF	17.8 ^b^	21.7 ^a^	16.5 ^b^	13.7 ^c^	13.1 ^cd^	10.9 ^de^	7.73 ^e^	
ABTS	CH	14.6 ^a^	12.7 ^a^	8.67 ^b^	8.20 ^b^	9.17 ^b^	7.72 ^b^	7.34 ^b^	0.424
	RF	13.8 ^a^	13.6 ^a^	8.20 ^b^	8.80 ^b^	9.08 ^b^	7.23 ^b^	7.16 ^b^	
FRAP	CH	227.9 ^a^	232.5 ^a^	191.6 ^b^	162.5 ^c^	167.7 ^c^	165.8 ^c^	158.1 ^c^	1.593
	RF	218.3 ^b^	231.7 ^a^	192.9 ^b^	164.1 ^c^	171.3 ^c^	159.4 ^c^	160.5 ^c^	

Abbreviations: M: mean; RMSE: Root Mean Standard Error; TPC: Total Phenolic Content (mg Gallic acid 100 mL^−1^); DPPH: 2,2-diphenyl-1 picrylhydrazyl radical-scavenging activity (mg Trolox 100 g^−1^); ABTS: 2.2′-Azinobis-3-Ethylbenzothiazoline-6-Sulfonicradical Cation Decolouration Assay (mg Trolox 100 g^−1^); FRAP: Ferric Reducing Antioxidant Power Assay (mg Fe^2+^ 100 mL^−1^). ^a–e^ Means with different superscripts are different for *p* < 0.05 (Two-way ANOVA; Tukey Test).

## Data Availability

Data are contained within the article.
